# Thalamic Nucleus Reuniens Glutamatergic Neurons Mediate Colorectal Visceral Pain in Mice via 5-HT_2B_ Receptors

**DOI:** 10.1007/s12264-024-01207-0

**Published:** 2024-05-13

**Authors:** Di Li, Han Du, Shu-Ting Qu, Jing-Lai Wu, Yong-Chang Li, Qi-Ya Xu, Xia Chen, Xiao-Xuan Dai, Ji-Tian Xu, Qian Wang, Guang-Yin Xu

**Affiliations:** 1 Jiangsu Key Laboratory of Neuropsychiatric Diseases and Institute of Neuroscience, Soochow University, Suzhou, 215123 China; 2 Department of Gastroenterology, The First Affiliated Hospital of Soochow University, Suzhou, 215123 China; 3 Department of Anesthesiology, Children’s Hospital of Soochow University, Suzhou, 215123 China; 4 Department of Physiology and Neurobiology, College of Basic Medical Sciences, Zhengzhou University, Zhengzhou, 450001 China

**Keywords:** Colorectal visceral pain, Thalamic nucleus reuniens, Neonatal maternal deprivation, Glutamatergic neurons, 5-HT_2B_ receptors

## Abstract

Irritable bowel syndrome (IBS) is a common functional bowel disorder characterized by abdominal pain and visceral hypersensitivity. Reducing visceral hypersensitivity is the key to effectively relieving abdominal pain in IBS. Increasing evidence has confirmed that the thalamic nucleus reuniens (Re) and 5-hydroxytryptamine (5-HT) neurotransmitter system play an important role in the development of colorectal visceral pain, whereas the exact mechanisms remain largely unclear. In this study, we found that high expression of the 5-HT_2B_ receptors in the Re glutamatergic neurons promoted colorectal visceral pain. Specifically, we found that neonatal maternal deprivation (NMD) mice exhibited visceral hyperalgesia and enhanced spontaneous synaptic transmission in the Re brain region. Colorectal distension (CRD) stimulation induced a large amount of c-Fos expression in the Re brain region of NMD mice, predominantly in glutamatergic neurons. Furthermore, optogenetic manipulation of glutamatergic neuronal activity in the Re altered colorectal visceral pain responses in CON and NMD mice. In addition, we demonstrated that 5-HT_2B_ receptor expression on the Re glutamatergic neurons was upregulated and ultimately promoted colorectal visceral pain in NMD mice. These findings suggest a critical role of the 5HT_2B_ receptors on the Re glutamatergic neurons in the regulation of colorectal visceral pain.

## Introduction

IBS is a functional gastrointestinal disorder characterized by chronic visceral pain. About 20% of the global population suffers from IBS [1]. Visceral pain signals reinforce certain neural circuits through peripheral and central sensitization, culminating in chronic visceral hypersensitivity [2, 3]. Despite some advances, the treatment of chronic visceral pain remains a major challenge for patients with IBS. As a result, there is an urgent need for researchers and clinicians to figure out its pathogenesis. Neonatal maternal deprivation (NMD) is a well-established animal model that induces chronic visceral hypersensitivity in rodents [4–6]. This study aims to investigate the central neural mechanisms underlying NMD-induced chronic visceral hypersensitivity in mice.

The thalamic nucleus reuniens (Re) is a key part of the midline thalamic nuclei. It has been reported that Re is involved not only in pain but also in fear, anxiety, depression, inflammation, and so on [7–9]. Peripheral nociceptive signals are transmitted to the central nervous system (CNS) *via* the dorsal root ganglia and the spinal cord, and the thalamus relays these signals to the cortex for pain perception. It has been reported that cortical nucleus groups such as the anterior cingulate (ACC), insula cortices (IC), and prefrontal cortex (PFC) play important roles in chronic visceral pain [10, 11]. Efferent projections of the Re are observed in many brain regions, including ACC, inferior limbic (IL) and anterior limbic (PL), medial orbital cortex (MO), and ventral orbital cortex (VO), while the Re also receives projections from other brain regions, such as ACC, mPFC, IC, CA1 region of hippocampus (HIP), zona incerta (ZI), periaqueductal gray matter (PAG), and ventral tegmental area (VTA) [12, 13]. Therefore, the Re may be a key brain region for pain signal relay from peripheral to cortex, and a very important pathway hub for descending transmission from central to peripheral in response to pain. However, studies on the involvements of the Re in visceral pain are very limited, so the mechanism of its modulation on chronic visceral pain still needs to be investigated.

The Re receives projections from serotonergic neurons in pain-related brain regions such as the dorsal raphe nucleus (DRN) and raphe magnus (RM) [14–16]. 5-HT is an important neurotransmitter in the body, involved in many physiological and pathological processes, and plays a crucial role in the processing and regulation of chronic pain in the CNS [17]. It has been consistently shown that the brain expresses 7 families of 5-HT receptors (5-HT_1-7_), including 14 subtypes in total [18]. Most 5-HT receptors exist in the brain and peripheral tissues, except for 5-HT_1E_, 5-HT_2C,_ and 5-HT_6_ receptors, which have limited expression in the central nervous system. The 5-HT_2_ receptors family consists of the 5-HT_2A,_ 5-HT_2B_, and 5-HT_2C_ receptors subtypes.

Increased excitatory postsynaptic currents in neurons mediated by 5-HT_2_ receptors have often been observed in brain slices, possibly because increased glutamate neurotransmitter release directly or indirectly mediates potassium channel closure and depolarization [19, 20]. Activation of the 5-HT_2B_ receptors enhanced the excitability of spinal dorsal horn neurons in the neuropathic pain model [21]. Moreover, the activation of 5-HT_2B_ receptors promotes contraction of the intestinal smooth muscle [22]. Therefore, inhibiting 5-HT_2B_ may reduce excessive intestinal contractions, subsequently relieving visceral pain caused by intestinal spasms. The function of the 5-HT_2B_ receptors in the brain remains largely unexplored.

In this study, we identified the Re as a key player in colorectal visceral pain in mice. Our findings revealed that Re glutamatergic neurons were activated during colorectal visceral pain processing and that manipulation of Re glutamatergic neurons altered chronic visceral pain behavior in mice. Furthermore, we demonstrated that increased 5-HT neurotransmission in the Re and expression of 5HT_2B_ receptors in glutamatergic neurons mediated the development of colorectal visceral pain. These findings will provide a new therapeutic target for the treatment of IBS.

## Materials and Methods

### Animals

All experiments were approved by the Soochow University Institutional Animal Care and Use Committee. All animals were housed at Suzhou University Experimental Animal Center, with a 12 h light-dark cycle, free access to food and water, a constant temperature of 23–25°C, and a relative humidity of 40%–60%. All methods employed in the study strictly adhered to the guidelines established by the International Association for the Study of Pain. Mice were randomly assigned to experimental and control groups.

### Neonatal Maternal Deprivation

The male pups were separated from their mothers for 3 h every day, from day 2 to day 15 after birth, by transferring into a clean mold box with an electric blanket. The mothers were left in the original cages when separated. A partition was placed in the mold box to ensure each pup was placed in a separate compartment. The pups isolated from the mother during the neonatal period were model group mice (NMD). Male offspring mice aged 6–12 weeks were selected for subsequent experiments [23, 24].

### Extracellular Recording

For acute extracellular recording, a single electrode was implanted into the Re (AP: −0.7 mm, ML: −0.2 mm, DV: −4.25 mm). The mice were allowed to recover for 7 days before recordings. Recording electrodes were attached to a 16-channel head-stage, and neuronal signals were amplified, filtered at a bandwidth of 300–5000 Hz, and stored using AlphaLab SNR (Alpha Omega, Jerusalem, Israel). Neuronal firings in the Re were recorded in the CON group and NMD group (lasted for 20 s). The firing rates were calculated using Neuroexplorer 5 (Nex Technologies, USA).

### Colorectal Visceral Pain Threshold Test

Colorectal visceral pain threshold was evaluated by CRD as previously described [24–26]. After the mice were anesthetized, the prepared balloon was slowly inserted 2 cm into the colorectum and secured to the tail [27]. The mice were restrained in a transparent resin box. While inflating the latex balloon, we recorded the pressure value at which the mouse's abdomen lifted off the tabletop as the colorectal visceral pain threshold [23]. All behavioral tests were conducted in a double-blinded manner.

### Electromyography Recording

Mice were completely anesthetized with isoflurane and electrodes were implanted as previously described [24]. Briefly, mice were kept on a thermostatic electric blanket, and the abdominal skin was cut open to thread the electrodes through the muscles on both sides of the mid-abdomen. The electrodes were threaded subcutaneously and pulled out from their neck. Finally, the exposed wires were folded together and sealed with parafilm to prevent mouse biting. EMG responses to CRD of different pressures were measured after a 7-day recovery period. The EMG signals are amplified and filtered at 300 Hz, followed by digitization using Acknowledge software (Biopac Systems, Inc). Computer algorithms were employed to calculate the area under the curve (AUC) of EMG activity during each 20-second interval of CRD [24].

### Immunofluorescence

After allowing mice to acclimate within transparent resin boxes for 1 h, CRD stimulation was given at 60 mmHg for 5 times, each lasting 20 s with a 4 min interval. 1.5 h after CRD stimulation, mice underwent cardiac perfusion to facilitate brain tissue collection, which was then fixed with 4% paraformaldehyde for 3 h. The brain tissue was sliced into 30 μm sections using a cryostat (Leica, CM3050 S, Germany). These slices were stored at −30°C for subsequent immunofluorescent staining. In the c-Fos assessment experiment involving local drug injection, NMD mice were administered RS-127445 (1 μL, 30 μmol/L) 1 h before CRD stimulation (20 s, 60 mmHg).

Initially, sections were incubated with 3% Triton X-100 and blocked with 10% donkey serum, prior to incubation with primary antibodies including mouse 5-HT_2B_ (1:200, Santa Cruz Biotechnology), rabbit anti-c-Fos (1:200, Cell Signaling, USA), rabbit anti-Glutamate (1:200, Cell Signaling Technology, USA) overnight in the fridge. After washes with 1x PBS buffer, sections were incubated for 1 h with appropriate secondary antibodies including anti-rabbit Alexa Fluor 488 (1:500, Invitrogen, USA) or anti-mouse Alexa Fluor 555 (1:500, Invitrogen, USA). Following 1xPBS washing, sections were air-dried and mounted using a mounting medium containing DAPI (4′,6-diamidino-2-phenylindole). Finally, laser confocal microscopy was employed to capture images of interest.

### Stereotaxic Injection

The mice were deeply anesthetized with isoflurane and fixed horizontally on a brain stereotaxic localizer, and the fur was cut to expose the skull (RWD, 71000-M, China). The bregma was used as the origin and then the cranial vertex plane to the horizontal position. The electrode tip was moved to the position of the Re, and the skull drill was used to drill holes in the skull (AP: −0.7 mm; ML: −0.2 mm; DV: −4.25 mm). 200 nL of the virus (rAAV2/9-VGluT2-hChR2-EYFP, rAAV2/9-VGluT2-eNpHR-EYFP or rAAV2/9-VGluT2-EGFP from Gene Biotechnology, China) was injected at a rate of 20 nL/min. The needle was kept in situ for 10-15min after each injection. After injection, a fiber optic cannula was inserted into the injection site and secured by dental adhesive. 3 weeks were given prior to testing to allow virus expression [28].

### Local Drug Infusion

Cannulas were embedded in the Re 7 days prior to the injection of 5-HT_2B_ receptors antagonist RS-127445 (3 μmol/L, 10 μmol/L, 30 μmol/L, 100 μmol/L, 1 μL) into the right Re at a flow rate of 0.2 μL/min. Similarly, vehicle solution (DMSO, 1 μL) was locally applied. The injector was slowly withdrawn for 1 min and the behavioral assays were performed roughly 15 min after the infusion. Unless otherwise stated, all drugs were purchased from MCE [29].

### Optogenetics

The ceramic inserts (diameter: 200 μm, Newdoon, China) were implanted into the Re of anesthetized mice using a stereotaxic apparatus. These ceramic inserts were secured to the mice’s skull using dental cement. The implanted ceramic inserts were connected to a laser generator through optical fiber tubing. Blue light (473 nm, 2–5 mW, 20 ms pulses, 10 Hz) or yellow light (589 nm, 3–5 mW, constant) was controlled by STSI-Optogenetics-LED (Alpha Omega, Jerusalem, Israel). Following the conclusion of the experiments, all mice’s ceramic insertion points were inspected. If a mouse's insertion point was found to be outside of the Re, the corresponding data were discarded [16].

### Brain Slice Electrophysiology

Mice were anesthetized and their brain tissue was rapidly obtained following cardiac perfusion with artificial cerebrospinal fluid (ACSF) (in mmol/L: 93 NMDG, 1.2 NaH_2_PO_4_, 30 NaHCO_3_, 20 HEPES, 2.5 KCl, 2 thiourea, 3 sodium pyruvate, 5 sodium ascorbate, 10 MgSO_4_, 12 NAC, 0.5 CaCl_2_, 25 glucose). Coronal slices of the brain tissue with a thickness of 300 μm were prepared using a vibrating microtome (Leica, VT1200S) and then incubated in ACSF at 32°C for 30 min. The slices were placed on a nylon mesh, ensuring continuous perfusion of the recording chamber with an oxygenated external solution (in mmol/L: 124 NaCl, 1.2 NaH_2_PO_4_, 2.5 KCl, 5 HEPES, 24 NaHCO_3_, 12.5 glucose, 2 MgSO_4_, 2 CaCl_2_) throughout the experiment. First, the target brain region was examined under a 5× microscope objective using a patch-clamp system and then transferred to the 40x water-immersion objective (Olympus, Japan) with a CCD camera, equipped with infrared-differential interference contrast (IR-DIC) and an infrared camera connected to the video monitor. An appropriate amount of electrode fluid was injected into the electrode and positive pressure was applied to observe the electrode resistance (4–8 MΩ). The glass electrode was slowly lowered to the brain slice below the recording solution surface, positive pressure was released in bath voltage clamp mode, and then negative pressure was given in cell mode to break the membrane of the cells for whole-cell patch clamp recordings. Action potentials (APs) and resting membrane potential (RP) were recorded during the current clamp. Spontaneous excitatory postsynaptic currents (sEPSC) were recorded at −70 mV [30, 31]. To record the glutamatergic neurons in the Re, a virus was injected into Re to specifically label glutamatergic neurons with an enhanced green fluorescent protein (EGFP, Fig. 5G).

### Western Blotting

The Re sample was homogenized in tissue lysate according to the weight of the tissue [29, 32]. Ultrasonic crushing (PW = 5.0 W) was performed in the ice-water mixture until the sample became transparent. After crushing, the samples were placed on ice for 2 h and centrifuged at 15000 rpm at 4 °C for 30 min to absorb the supernatant. A BCA protein assay kit (Beyotime, China) was used to determine the protein concentration. The same amount of protein (30 μg) was subjected to 4% and 10% SDS-polyacrylamide gel electrophoresis (PAGE) and the protein bands were transferred to a nitrocellulose membrane (Merck Millipore, Germany). The membrane was incubated with the primary antibody (1:1000, anti-GAPDH; 1:50, anti-5HT_2B_) at 4 °C overnight. After TBST rinse and incubation with goat anti-Mouse IgG, enhanced luminescence (ECL, NCM biotech, China) and appropriate luminescence imaging system (Bio-Rad) were used to develop the image after washing the second antibody, and the gray values of protein bands were analyzed by ImageJ software. With GAPDH as a reference, the gray values of protein bands were standardized and then analyzed statistically [31].

### Real-Time PCR

Real-time polymerase chain reaction (PCR) was used to detect the mRNA expression of the 5-HT_2B_ receptors. The samples were mixed in the reverse transcription kit (Transgen Biotech, China) according to the manufacturer’s instructions. The extracted RNA was reverse-transcribed into cDNA using a PCR system. Then, the mRNA expressions of the 5-HT_2B_ receptors were detected by the real-time quantitative PCR (qPCR) reaction. Relative mRNA expression was analyzed using the obtained Ct value. The sequences of the primers for 5-HT_2B_ and GAPDH are shown below.

**Table:** 

Primer sequences used in the present study	
Primers	Sequences (5′ to 3′)
GAPDH-F	GAAGGTCGGTGTGAACGGAT
GAPDH -R	AATCTCCACTTTGCCACTGC
5-HT_2B_-F	ATGAAGCAGACTGTGGAGGG
5-HT_2B_-R	TCCAGTGCAACAGCCAGAAT
5-HT_1A_-F	TCCGCAAGACGGTCAAGAAG
5-HT_1A_-R	CTGTCTCACCGCCCCATTAG
5-HT_1B_-F	TGCTCCTCATCGCCCTCTAT
5-HT_1B_-R	CCTAGCGGCCATGAGTTTCT
5-HT_1D_-F	GCTCTCAGAATCTCGCTCGT
5-HT_1D_-R	GGTGGTAAGGACGAAGGCAT
5-HT_1F_-F	CTGTCTGGGCTGGCATTGAT
5-HT_1F_-R	TGCAGCTTCCGAGTCACAAT
5-HT_2A_-F	ACCATAGCCGCTTCAACTCC
5-HT_2A_-R	CCGAAGACTGGGATTGGCAT
5-HT_3B_-F	CTGGCCATCCTGTATCGCTT
5-HT_3B_-R	AGGTGACGGTATAGAGCCCC
5-HT_5A_-F	TTGGAACCTAACCGCAGCTT
5-HT_5A_-R	ACCAGGCTCAGTGGCATA AC
5-HT_5B_-F	CGCCGAGCAACAGTAACCTT
5-HT_5B_-R	CAACAAAGCACAAACACGCC
5-H_T6_-F	ACTTCTTCCTGGTGTCGCTC
5-H_T6_-R	AGAGGTTGAGAATGGAGGCG

### Statistical Analyses

All data were presented as Mean ± SEM. Statistical analyses were conducted using GraphPad Prism 8.0.2. Prior to statistical analysis, normality checks were performed on all data. Significance was determined using a two-tailed Student's *t*-test or one-way analysis of variance (ANOVA), followed by the Tukey test for multiple comparisons, or two-way ANOVA followed by the Bonferroni test for multiple comparisons. Differences were considered significant when *P <0.05.

## Results

### The Re Contributed to Visceral Hypersensitivity in NMD Mice

**Fig. 1:**
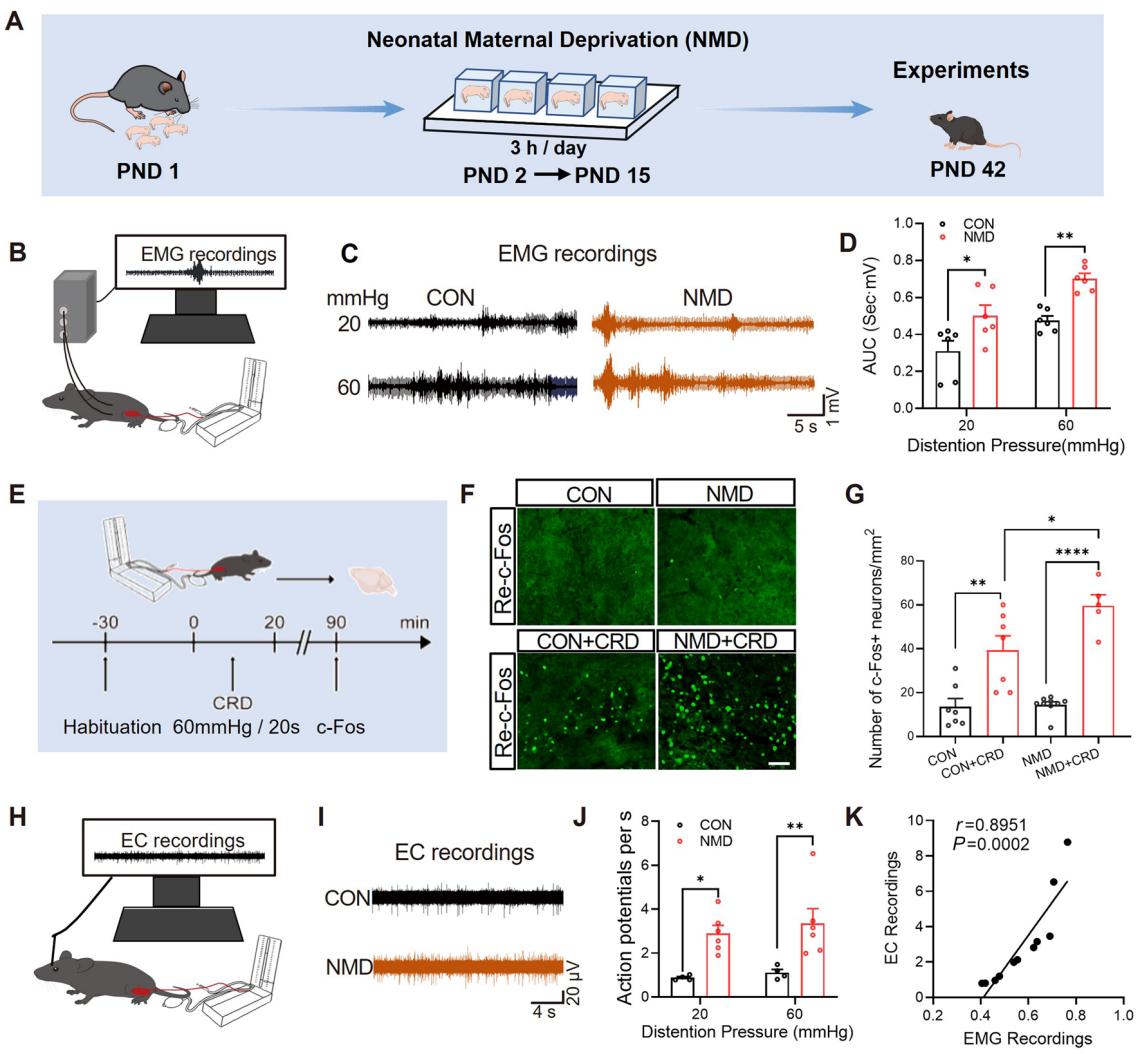
The Re was involved in colorectal visceral pain in NMD mice. **A** Experimental protocol for NMD model preparation. **B** Schematic of EMG recording. **C** Representative traces of EMG recording of the CON and NMD groups (CON, *n =* 6 mice; NMD, *n =* 6 mice, *P <0.05, **P <0.01, two-way ANOVA). **D** The bar graph shows the AUC of EMG recordings at the same distending pressure. **E** The experimental protocol of CRD stimulation. **F** Representative c-Fos immunofluorescence induced by CRD stimulation. Scale bar, 50 μm. **G** NMD enhanced c-Fos expression in response to CRD stimulation at the Re region (CON, *n =* 7 slices; NMD, *n =* 9 slices; CON+CRD, *n =* 7 slices; NMD+CRD, *n =* 5 slices, *P <0.05, **P<0.01, ****P <0.0001, one-way ANOVA). **H** Schematic diagram of EC recordings. **I** Representative traces of EC recording. **J** NMD increased Re neural firing (CON, *n =* 4 mice; NMD, *n =* 6 mice, *P <0.05, **P <0.01, two-way ANOVA). **K** Correlation analysis shows a positive correlation between EMG and extracellular recordings at 20 and 60 mmHg CRD stimulation.

We first evaluated the effect of maternal deprivation (as illustrated in Fig. 1A) on colorectal visceral pain hyperalgesia. These NMD mice were tested for colorectal visceral pain at 6 weeks of age. Abdominal wall electromyography (EMG) (Fig. 1B) showed that the AUC of EMG induced by CRD was enhanced in NMD mice (Fig. 1C, D), suggesting visceral hyperalgesia in NMD mice compared to CON mice. Next, c-Fos staining was used to explore which brain regions were activated by CRD (Fig. 1E). We found a large amount of c-Fos expression in the Re after CRD, and more importantly, the c-Fos expression in the NMD+CRD group was much more than that in the CON+CRD group (Fig. 1F, G). The results suggest that enhanced activation of neurons in the Re may contribute to visceral hyperalgesia in NMD mice. In addition, we performed an *in vivo* single-channel extracellular recordings (EC) test on the Re cells (Fig. 1H). Statistical analysis showed that the average discharge rate of Re cells in the NMD group was significantly higher than that in the CON group (Fig. 1I, J). Furthermore, the results of EMG recordings and EC recordings were positively correlated (Fig. 1K). These results confirmed our hypothesis that Re is involved in chronic visceral hypersensitivity in NMD mice.

### NMD Increased Neuronal Excitability and Synaptic Transmission in the Re

**Fig. 2:**
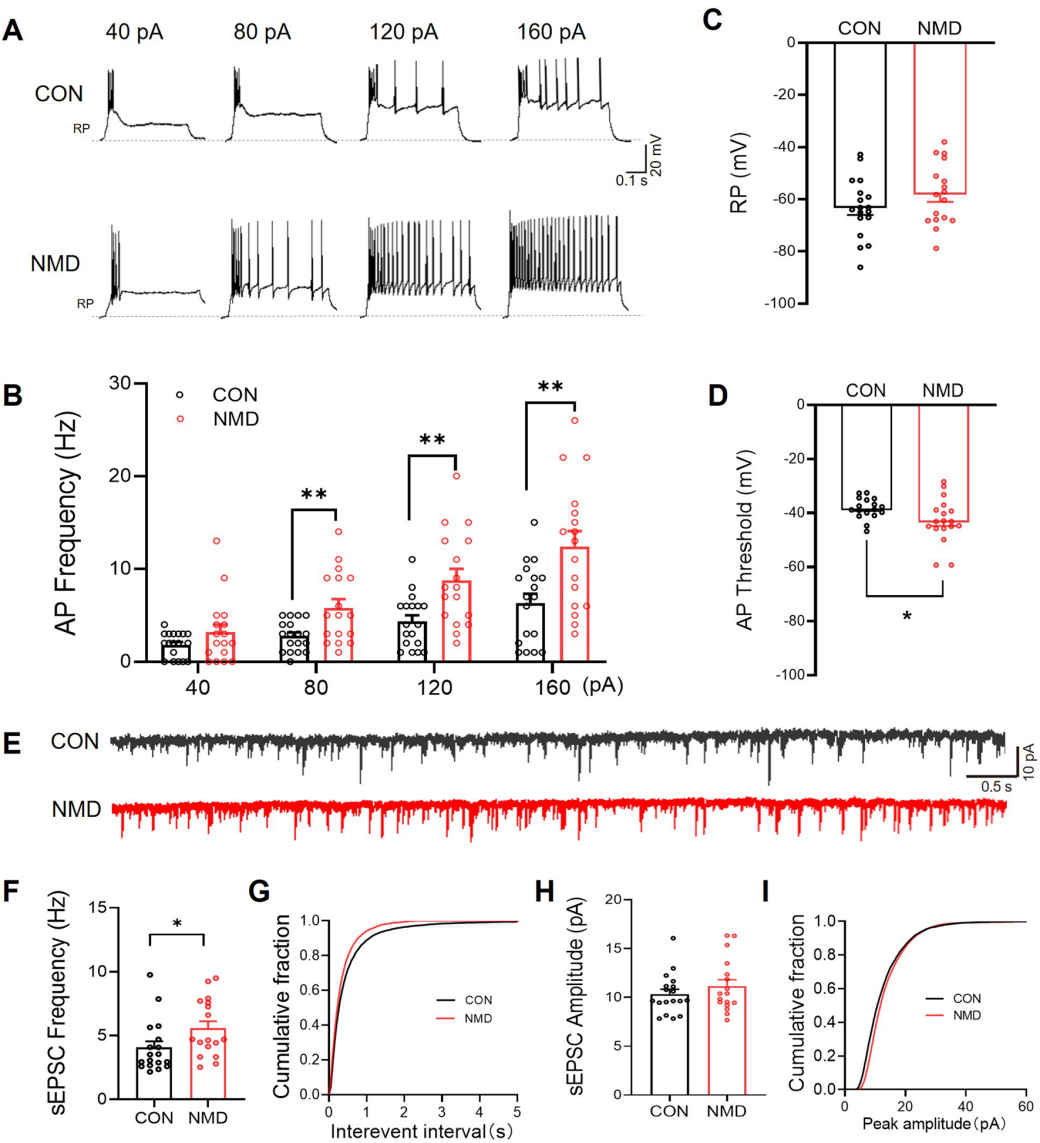
NMD increased neuronal excitability and synaptic transmission in the Re. **A** Representative traces of the current-evoked action potentials in Re brain slices of mice in the CON and NMD groups. **B** NMD increased the current-evoked action potentials in brain slices of NMD mice (CON, *n =* 18 cells; NMD: *n =* 17 cells, **P <0.01, two-way ANOVA). **C**, **D** Comparison of RP and AP threshold between CON and NMD mice (CON, *n =* 18 cells; NMD: *n =* 17 cells, *P <0.05, Student’s *t*-test). **E** Representative traces of sEPSC. **F**, **H** Statistics analysis of sEPSC frequency and amplitude in brain slices of mice in the CON and NMD groups (CON, *n =* 18 cells; NMD: *n =* 17 cells, *P <0.05, Student’s *t*-test).

In order to more comprehensively verify the involvement of Re in colorectal visceral pain, the whole-cell patch-clamp technique was used to assess the excitability of Re region neurons in CON and NMD mice (Fig. 2A). There was no significant difference in the resting membrane potential (RP) of the Re neurons in CON and NMD mice (Fig. 2C). The action potential (AP) thresholds of the Re neurons in NMD mice were significantly lower than in the CON group (Fig. 2D). In addition, the frequency of AP in Re neurons of NMD mice was significantly increased by 80 pA, 120 pA, and 160 pA current stimulation compared with CON mice (Fig. 2B). The above results suggest that the excitability of neurons in Re of NMD mice is significantly elevated, which may contribute to the development of visceral hypersensitivity in NMD mice.

Next, we used the whole-cell patch clamp voltage-clamp technique to record spontaneous excitatory postsynaptic currents (sEPSC) of neurons in the Re of CON and NMD mice. The sEPSC model shows enhanced excitatory synaptic transmission of neurons in the Re of NMD mice (Fig. 2E). Compared with the CON group, the sEPSC frequency of neurons in Re of NMD mice was significantly enhanced (Fig. 2F), while there was no significant change in sEPSC amplitude (Fig. 2H), indicating increased release of presynaptic excitatory neurotransmitter. The above results suggest that NMD induces visceral hypersensitivity in mice by enhancing presynaptic excitatory neurotransmitter release in the Re region.

### Glutamatergic Neurons in the Re Contributed to Visceral Hyperalgesia in NMD Mice

**Fig. 3:**
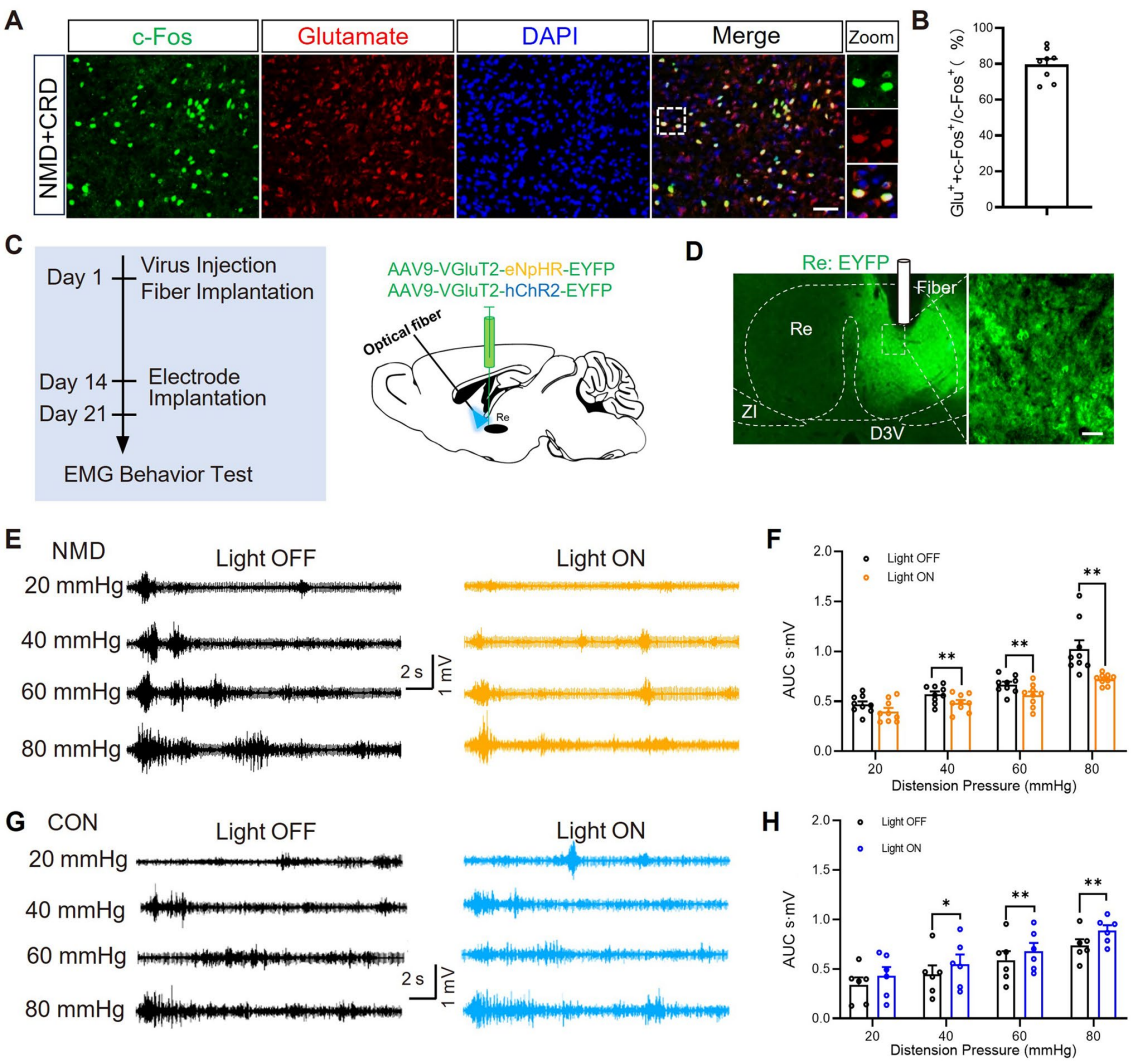
Excitatory glutamatergic neurons in the Re participated in colorectal visceral pain of NMD mice. **A** Representative images of c-Fos^+^ cells (green) and glutamatergic neurons (red) co-expression in the Re. Scale bar, 50 μm. **B** The percentage of c-Fos^+^ neurons that are glutamatergic (*n =* 8 slices). **C**, **D** Schematic of optogenetic experiments of CON and NMD mice. Scale bar, 20 μm. **E** Representative traces of EMG recordings in NMD mice. **F** Optogenetic inhibition of Re neurons inhibited colorectal visceral pain responses (eNpHR, CON, *n =* 9 mice; NMD, *n =* 9 mice, **P <0.01, two-way ANOVA). **G** Representative traces of EMG recordings in CON mice. **H** Optogenetic activation of Re neurons enhanced colorectal visceral pain responses (hChR2, CON, *n =* 6 mice; NMD, *n =* 6 mice, *P <0.05, **P <0.01, two-way ANOVA).

Next, we explored the neuron types of the Re neurons activated by CRD. In NMD mice, c-Fos-positive Re neurons were co-labeled with the marker of glutamatergic neurons (Fig. 3A). We found that about 80% of the CRD-activated neurons were glutamatergic neurons (Fig. 3B). It suggests that glutamatergic neurons of Re are involved in colorectal visceral pain in NMD mice. Subsequently, we manipulated the activity of glutamatergic neurons through optogenetics to examine the role of glutamatergic neurons in Re in the regulation of colorectal visceral pain. In NMD mice, inhibiting the activity of glutamatergic neurons in Re with yellow light (580 nm) effectively relieved colorectal visceral pain during CRD stimulation (Fig. 3E, F). In CON mice, activation of glutamatergic neurons with blue light (473 nm) significantly promoted colorectal visceral pain (Fig. 3G, H). The above results suggest that activation of glutamatergic neurons in Re promotes colorectal visceral pain in NMD mice.

### 5-HT_2B_ Receptors Expression in Re Glutamatergic Neurons and 5-HT Release in the Re were Increased

**Fig. 4:**
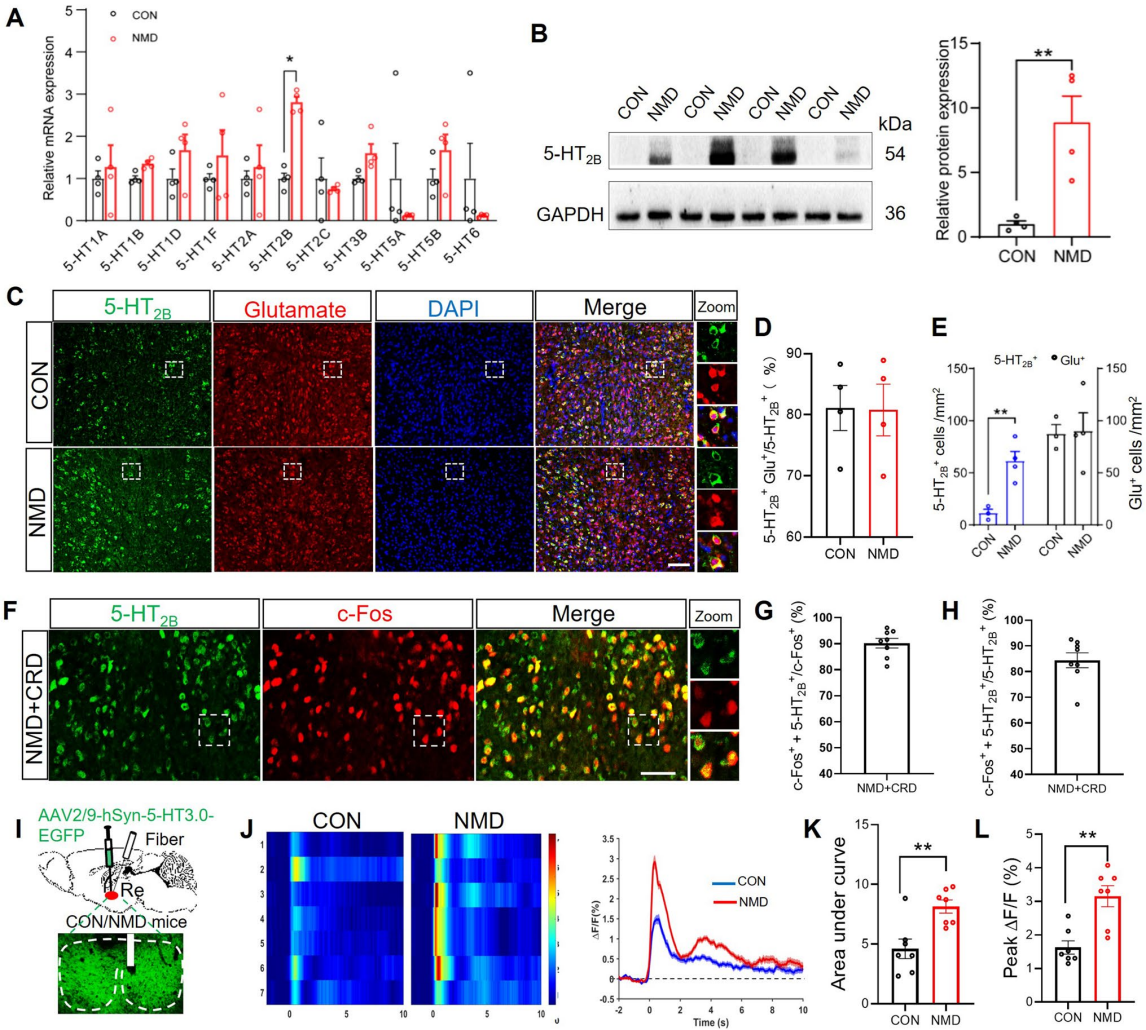
5-HT_2B_ and 5HT in the Re were increased in NMD mice. **A** mRNA expression levels of different 5-HT receptor subtypes in Re (CON, *n =* 4 mice; NMD, *n =* 4 mice, *P <0.05, two-way RM ANOVA). **B** The protein level of the 5-HT_2B_ receptors in Re was significantly higher in NMD mice compared with CON mice (CON, *n =* 4 mice; NMD, *n =* 4 mice, **P <0.01, Student’s *t*-test). **C** Representative images of 5-HT_2B_ reporters (green) and glutamatergic neurons (red) co-expression in the Re of NMD and CON mice. Scale bar, 50 μm. **D** Percentage of 5-HT_2B_^+^ neurons that are glutamatergic neurons in CON and NMD mice (CON, *n =* 4 slices; NMD, *n =* 4 slices). **E** The density of glutamatergic neurons and the number of 5-HT_2B_^+^ neurons in CON and NMD groups (CON, *n =* 4 mice; NMD, *n =* 4 mice, **P <0.01, Student’s *t*-test). **F** Representative images of 5-HT_2B_ reporters (green) and c-Fos (red) co-expression in the Re of NMD mice. Scale bar, 50 μm. **G** Percentage of c-Fos^+^ neurons that are 5-HT_2B_^+^ neurons (*n =* 8 slices). **H** Percentage of 5-HT_2B_^+^ neurons that are c-Fos^+^ neurons (*n =* 8 slices). **I** Schematic of 5-HT probe injection. Typical images of 5-HT probe injection sites within the Re. **J** Thermograms and event-related line plots of 5-HT changes in CON and NMD mice during 60 mmHg CRD pressure stimulation. **K** Statistical histograms of the area under the curve (AUC) of 5-HT release in CON and NMD mice during CRD pressure stimulation at 60 mmHg (CON, *n =* 6 mice; NMD, *n =* 6 mice, *P <0.05, **P <0.01, Student’s *t*-test). **L** Statistical histograms of peak 5-HT release in CON and NMD mice during CRD pressure stimulation at 60 mmHg (CON, *n =* 6 mice; NMD, *n =* 6 mice, *P <0.05, **P <0.01, Student’s *t*-test).

To further verify the molecular mechanism by which glutamatergic neurons in Re regulate colorectal visceral pain behaviors, the expression of 5-HT receptors was next evaluated. Among the subtypes examined, the mRNA level of 5-HT_2B_ receptors in the Re of NMD mice was significantly increased (Fig. 4A) and Western Blot further confirmed this result (Fig. 4B). To examine which type of neurons express the 5-HT_2B_ receptor, we co-labeled 5HT_2B_ and glutamate by immunofluorescence and found that about 80% of 5-HT_2B_ positive cells were glutaminergic neurons (Fig. 4C, D). The density of 5-HT_2B_ positive neurons in NMD mice was much higher than that in CON mice (Fig. 4E), whereas the number of glutamate-positive neurons had no significant change (Fig. 4E). In addition, approximately 90% of c-Fos^+^ neurons were also positive for 5-HT_2B_ (Fig. 4F, G), while around 85% of 5-HT_2B_^+^ neurons were c-Fos^+^ (Fig. 4H). This indicates a substantial co-labeling between c-Fos and 5-HT_2B_.

5-HT is an important neurotransmitter associated with pain. We used a probe specific for 5-HT to detect 5-HT release in the Re (Fig. 4I). Upon administration of CRD, both CON and NMD mice showed increased release of 5-HT in the Re (Fig. 4J). Statistical analysis showed that the AUC and peak values of the NMD group were significantly higher than those of the CON group (Fig. 4K, L), suggesting a higher release of 5-HT in NMD mice. These results suggested that the expression of 5-HT_2B_ receptors on Re glutamatergic neurons and 5-HT release in the Re were up-regulated in NMD mice.

### Specific Inhibition of 5-HT_2B_ Alleviated Colorectal Visceral Pain in NMD Mice

**Fig. 5:**
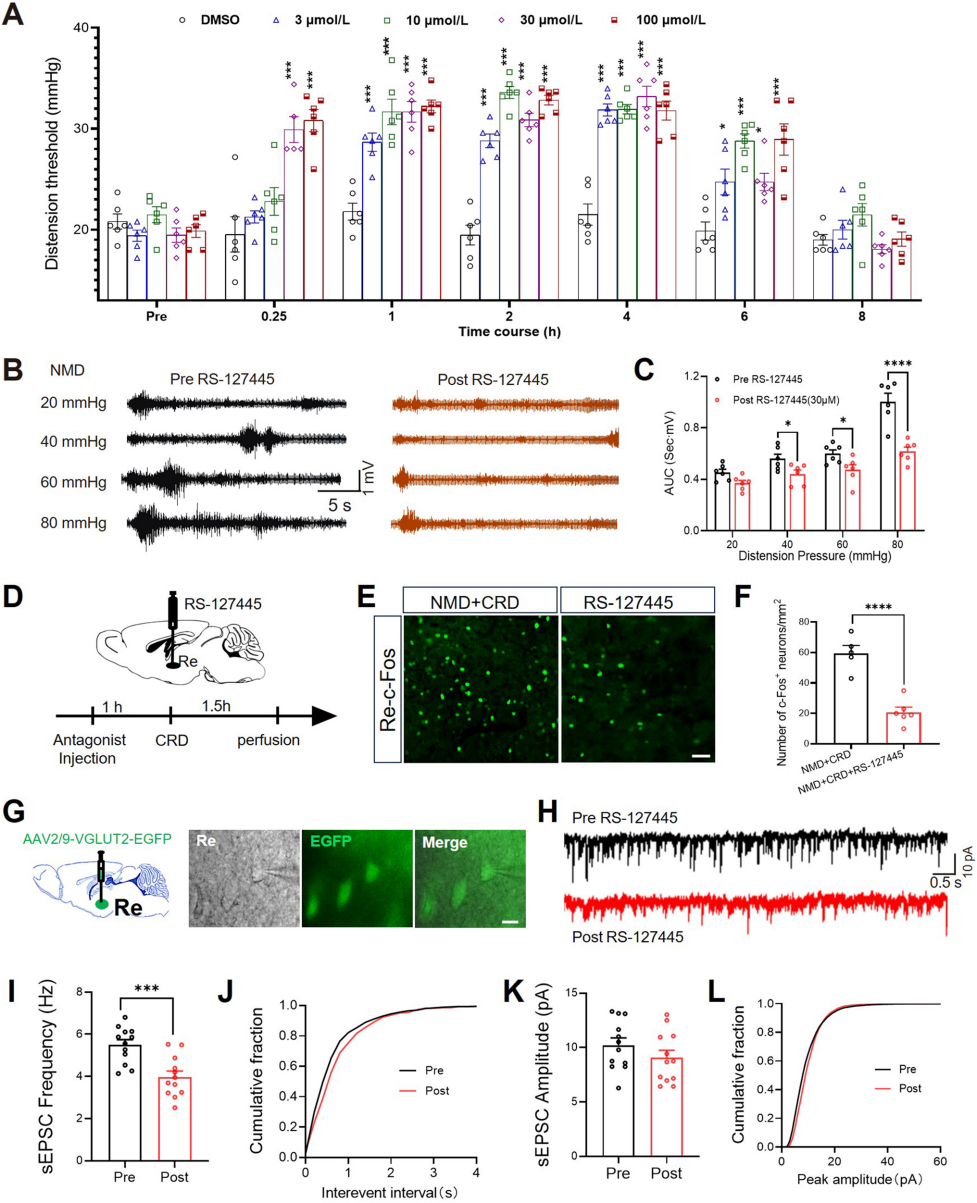
Inhibition of 5-HT_2B_ receptors relieved colorectal visceral pain in NMD mice. **A** Effects of different doses of 5-HT_2B_ receptors inhibitors on colorectal visceral pain thresholds in NMD mice (NMD=6 mice, *P <0.05, ***P <0.001, two-way ANOVA). **B** Representative traces of EMG recordings in NMD mice. **C** RS-127445 inhibited EMG responses. (NMD, *n =* 6 mice, *P <0.05, ****P <0.0001, two-way ANOVA). **D** Schematic time course of RS-127445 injection and brain harvest on NMD mice. **E** Representative immunofluorescence of c-Fos (green). Scale bar, 50 μm. **F** Statistical graph of c-Fos immunofluorescence in NMD+CRD and NMD+CRD+RS-127445 groups (NMD+CRD, *n =* 5 slices; NMD+CRD+RS-127445, *n =* 6 slices, ****P <0.0001, two-sample *t* test). **G** Representative images of real-time recordings of glutaminergic neurons in the Re. Scale bar, 10 μm. **H** Representative trace of sEPSC before and after incubation of RS-127445 in Re neurons of NMD neurons. **I**, **K** Statistics analysis of sEPSC frequency and amplitude before and after incubation of RS-127445 in Re neurons (NMD, *n =* 12 cells; NMD+RS-127445, *n =* 12 cells, ***P <0.001, Student’s *t*-test).

To further investigate the role of 5-HT_2B_ in colorectal visceral pain in NMD mice, we microinjected 1 μL DMSO and different doses (3 μmol/L, 10 μmol/L, 30 μmol/L, 100 μmol/L) of 5-HT_2B_ receptors antagonist RS-127445 into the Re of NMD mice, respectively, and examined the colorectal visceral pain threshold of the mice at different time course (0.25 h, 1 h, 2 h, 4 h, 6 h, 8 h). Doses of 3 μmol/L and 10 μmol/L exerted analgesic effects 1 h to 6 h after administration, and 30 μmol/L and 100 μmol/L exerted analgesic effects 0.25 h to 6 h after administration (Fig. 5A). Therefore, a dose of 30 μmol/L was selected for the following study. Furthermore, we assessed colorectal visceral pain by EMG recording (Fig. 5B). Inhibition of 5-HT_2B_ significantly reduced the AUC of EMG during CRD (Fig. 5C), that is, relieved colorectal visceral pain in NMD mice, indicating 5-HT_2B_ as a molecule accounting for colorectal visceral pain in NMD mice.

**Fig. 6:**
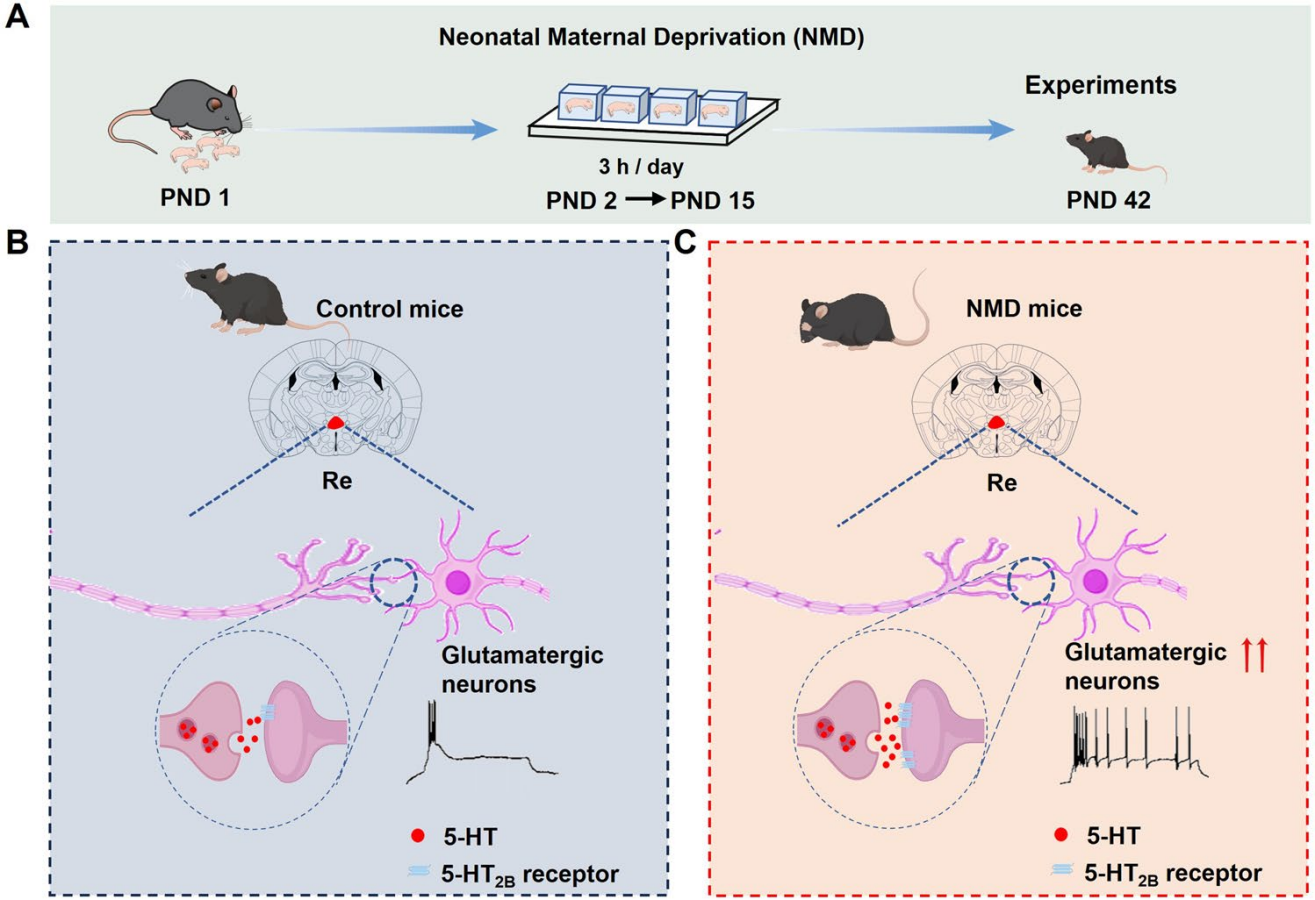
Schematic model of 5-HT_2B_ receptors-mediated colorectal visceral pain in Re glutamatergic neurons. **A** Schematic of the NMD Mouse Model Establishment Method. **B** Compared to CON mice, CRD induces increased firing of Re glutamatergic neurons in NMD mice, along with higher levels of 5-HT release, increased expression of 5-HT_2B_ receptors, and enhanced visceral pain responses in NMD mice.

In addition, we explored the effect of the 5-HT_2B_ antagonist (RS-127445) on c-Fos expression in NMD mice (Fig. 5D). CRD-induced c-Fos expression was significantly reduced in the group injected with RS-127445 in the Re (Fig. 5E, F). To clarify whether RS-127445 affects synaptic transmission of glutaminergic neurons in the Re, we recorded sEPSC of glutamatergic neurons from Re brain slices before and after incubation with RS-127445 by whole-cell patch clamp technique (Fig. 5G, H). The sEPSC frequency of glutamatergic neurons in Re of NMD mice was significantly reduced after incubation with RS-127445 (Fig. 5I), but the amplitude did not change (Fig. 5K), suggesting that the 5-HT_2B_ receptors contributed to visceral hyperalgesia in NMD mice (Fig. 6).

## Discussion

Colorectal visceral pain is one of the most common symptoms of gastrointestinal diseases. One of the possible causes of colorectal visceral pain is the excessive excitation of central neurons that receive synaptic input from visceral organs, resulting in aberrant downward modulation of pain transmission. Current treatments for patients with colorectal visceral pain, such as IBS, are far from satisfactory [33], and the precise mechanism behind the central nervous system's regulation of colorectal visceral pain remains to be elucidated. Experiences of trauma and neglect early in life permanently affect the function of the hypothalamic axis and are closely associated with colorectal visceral pain [34–40]. Therefore, we used the male NMD model to simulate human visceral pain and study the neural mechanism of colorectal visceral pain. The reason for not selecting female mice in the present study is that the secretion of estrogen in female mice may influence the perception of pain [41–43]. There are some studies indicating that the pain sensation in male and female mice may be regulated by different neurotransmitter systems, potentially necessitating distinct pharmacological approaches to treat pain in both sexes [44]. The regulation of pain by 5-HT receptors may exhibit gender differences [43], as reports indicate that the impact of serotonin neurotransmitters on pain in male mice is greater than in females [44]. Moreover, in a mouse model of acute thermal nociception, inhibiting 5-HT_2A_ receptors enhanced the potency of opioid drugs in male mice but not in females [45]. Examples like these lead us to speculate that the role of 5-HT_2B_ in Re may differ between male and female mice.

Previous studies have shown that the Re is related to pain, including participating in the analgesic effect of ketoprofen and opioid receptor 1 [46, 47], and it has been proved that the spinal cord is closely involved in the input and processing of pain. Typically, the thalamus serves as the first relay station for spinal cord input through the spinothalamic tract. Re nucleus is an integral component of the thalamus [48], suggesting that Re plays a very important role in pain. Re is associated with nociceptive perception and has also been shown to be activated in pain fMRI studies [47]. Our results showed that activation of Re neurons contributed to colorectal visceral pain in NMD mice. In our study, CRD induced a massive activation of glutamatergic neurons in Re leading to nociceptive hypersensitivity in NMD mice. Since not all of the Re glutaminergic neurons were activated after CRD stimulation, it is difficult to exclude the effect on behavior after other excitatory neurons are activated or inhibited in the optogenetic experiments. In this study, the majority of glutamatergic neurons in the Re activated by CRD stimulation were associated with visceral pain. The approximately 20% of glutamatergic neurons that remained unactivated may not participate in regulating visceral pain. Thus, even modulating this 20% of unactivated glutamatergic neurons may not affect our results. Admittedly, the reviewer raised an interesting question that deserves further exploration in future studies. In addition, electrophysiological results revealed that the excitability and synaptic transmission in the Re neurons were significantly higher than that in CON mice, which also indicated that Re was involved in colorectal visceral pain in NMD mice. Previous studies in our laboratory have proved that brain nuclei such as ACC, IC, and BLA are involved in colorectal visceral pain in NMD [23, 30, 31]. Future studies should investigate the connections between these brain regions and the Re to provide potential new targets for treating colorectal visceral pain.

5-HT, a monoamine neurotransmitter, has an important modulatory role in pain perception. We found a significant increase in 5-HT neurotransmission in Re during CRD. The 5-HT receptor is essential for chronic pain in the CNS [49, 50]. In fact, studies have proved that the 5-HT_2B_ receptor is involved in the central and peripheral regulation of colorectal visceral pain [51–53], but relevant studies are limited to the pharmacological level. It is not completely understood whether the level of 5-HT_2B_ receptor expression in the brain changes in NMD or how it regulates colorectal visceral pain. In this study, we confirmed that mRNA levels and protein levels of 5-HT_2B_ receptors were significantly increased in glutamatergic neurons in the Re. Next, inhibition of 5-HT_2B_ in Re not only reduced the expression of c-Fos but also alleviated colorectal visceral pain. Also, inhibition of 5-HT_2B_ in isolated brain slices decreased synaptic transmission frequency in Re. The specific mechanism underlying this result remains unclear. It is possible that there may be a form of indirect regulation. In a recently published study, the postsynaptic glutamate receptor mGlu5 affected presynaptic glutamate release and sEPSC frequency but not sEPSC amplitude by targeting mir-501-3p, a microRNA that regulates the release of presynaptic glutamate [54]. Therefore, we speculate that the postsynaptic 5-HT_2B_ receptors may modulate sEPSC frequency by indirectly regulating the release of presynaptic 5-HT in our study. These data suggest that NMD induces upregulation of 5-HT_2B_ receptors in glutamatergic neurons in the Re, increasing synaptic transmission frequency and, ultimately visceral hypersensitivity in NMD mice.

In conclusion, our study demonstrated that up-regulation of 5-HT_2B_ receptor expression on the Re glutamatergic neurons mediates colorectal visceral pain in NMD mice. The study elucidated, for the first time, the involvement of the Re glutamatergic neurons in colorectal visceral pain and updated the role of brain 5-HT_2B_ receptors in colorectal visceral pain. The 5-HT_2B_ receptor antagonist RS-127445 has a relieving effect on early-life stress-induced visceral hypersensitivity, which would provide new strategies for targeting Re as well as 5-HT_2B_ receptors for the treatment of colorectal visceral pain.
